# The Interaction between Water Currents and Salmon Swimming Behaviour in Sea Cages

**DOI:** 10.1371/journal.pone.0097635

**Published:** 2014-05-15

**Authors:** David Johansson, Frida Laursen, Anders Fernö, Jan Erik Fosseidengen, Pascal Klebert, Lars Helge Stien, Tone Vågseth, Frode Oppedal

**Affiliations:** 1 Institute of Marine Research, Matredal, Norway; 2 Department of Biology, University of Bergen, Bergen, Norway; 3 Sintef Fisheries and Aquaculture, Trondheim, Norway; Pacific Northwest National Laboratory, United States of America

## Abstract

Positioning of sea cages at sites with high water current velocities expose the fish to a largely unknown environmental challenge. In this study we observed the swimming behaviour of Atlantic salmon (*Salmo salar* L.) at a commercial farm with tidal currents altering between low, moderate and high velocities. At high current velocities the salmon switched from the traditional circular polarized group structure, seen at low and moderate current velocities, to a group structure where all fish kept stations at fixed positions swimming against the current. This type of group behaviour has not been described in sea cages previously. The structural changes could be explained by a preferred swimming speed of salmon spatially restricted in a cage in combination with a behavioural plasticity of the fish.

## Introduction

Moving sea cages to exposed sites with strong water currents is an industry-wide trend in Atlantic salmon (*Salmo salar* L.) farming [1], [2]. This could improve production efficiency through access to high water quality due to rapid transport and dilution of waste products, more stable temperatures, high levels of oxygen and less influence from terrestrial runoff [1], [3]. Other positive effects such as reduction of possible conflicts with other users in the coastal area and avoidance of the ecological carrying capacity limitations of inshore waters have been suggested [3]. One prerequisite for this progress has been the development of strong, resistant farm structures that can withstand the forces produced by strong water currents [4], [5]. However, it is not known how the fish inside the sea cages cope with high water current velocities. The fish has to cope with being forced into an environment that radically differs from the sheltered fjord sites. The question about the amount and type of stress produced by a high-energy environment and the fish capacity to cope is at least as important as the development of new resistant farming platforms. Salmon farms in sheltered localities generally experience current velocities below 20 cm s^−1^ outside the cages [6]. At such velocities the fish will often form a circular, one way directed uniform swimming pattern, possibly as a result of individuals actively avoiding collisions with each other and the cage wall [7]. At these sites salmon typically swim at speeds of 0.3–0.9 BL s^−1^, with maximum average values of 1.9 BL s^−1^ [8], [9], [10]. The constant swimming of salmon under natural conditions has been associated with an inherent migratory tendency related to optimum cruising speed [10] [11] and in open ocean studies the speed approximates to 1 BL s^−1^, independent of age [12]. Studies using swim tunnels indicate a critical swimming speed, U_crit_, for small salmon (400–800 g) of 1.6–2.2 BL s^−1^ [13], [14], although one study reports values as high as 3.0 BL s^−1^ [15].

Although the exact swimming capacity of salmon is uncertain, and will vary with such factors as size, exercise level, degree of satiation [16] and individual fitness, it is evident that salmon inside sea cages must adapt their behaviour to the water current. Hence the objective of this study was to observe the general effects of high water current velocities on fish swimming behaviour at the group level, in an exposed commercial salmon cage.

## Materials and Methods

**Figure 1:**
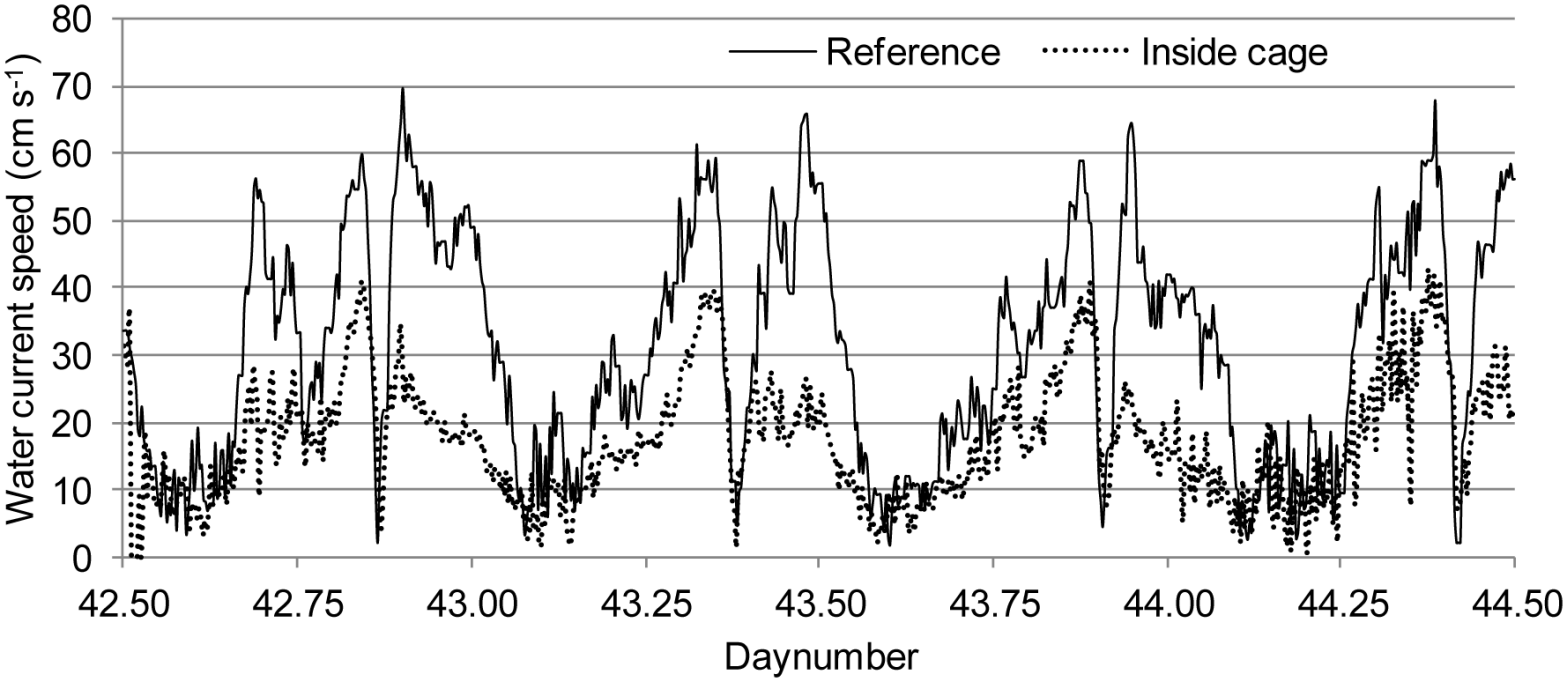
Water current velocity outside the cage (Reference) and inside the cage from 11th to 13th of February, 2012.

The observations of schooling behaviour were performed from 11^th^ to 13^th^ of February 2012 at a commercial marine salmon farm near Torshavn in the Faroe Islands, Denmark (61.59° N). The farm had 8 circular cages of 41 m diameter, and 2 cages of 50 m diameter, with a depth of 12 m to the bottom ring. The depth below the cages varied from 30 to 40 m, and the total biomass at site was 1320 tonnes. The fish were fed continuously from 08∶30 to 16∶15 h and were exposed to continuous artificial light at 4 m depth. The observed cage (41 m diameter) was selected based on having the highest probability to be exposed to high water current velocities, due to its position at the south end of the farm. According to farm data, the stocking density in this cage was 6.2 kg m^−3^ and the average fish weight 1.54 kg, corresponding to an approximate fish length of 50 cm. During the observation period, vertical profiles of water characteristics (oxygen, temperature and salinity) showed little spatial and temporal variation: dissolved oxygen saturation levels were at 94.6±2.3% (mean±SD), temperature 6.6±0.1°C and salinity 35.0±0.1 ppt, all of which were within accepted optimal limits [6], [17], [18]. Vertical profiles of water current down to 20 m of depth were recorded 210 m south of the farm with open sea between the observed cage and the reference point using an Acoustic Wave And Current profiler (AWAC, Nortek, Oslo, Norway). In order to minimize disturbance from the fish, single point measurements were taken at 6.2 m depth in the centre of the cage using a Vector Aquadopp 3D (Nortek, Oslo, Norway). The observed water current velocities varied in a tidal pattern between 0 to 69 cm s^−1^ at the reference point, and between 0 to 42 cm s^−1^ at the single point measured inside the cage (Figure 1). The reduced current velocity inside the cage (Figure 1) is related to dampening by the net and the fish inside the cage and the cages north of the observed cage [6], [19], [20]. The vertical profiles showed little differences in current speeds and directions between 0 and 12 m depth. The tidal nature of the current produced a variable main direction between 120° and 300°. Unless otherwise specified, we refer to the current data collected at the reference point. The schooling behaviour of the salmon was observed with two remotely controlled underwater pan/tilt cameras (Orbit GMT AS, Førresfjord, Norway) connected to a recording DVD player. One camera was positioned next to the net and the other was positioned approximately 15 m from the net at the opposite side of the cage at approximately 6 m of depth to give a good representation of behaviours both up- and downwards. The 48 h period of recordings were divided into four minutes subsamples, which were post-analysed and manually classified for swimming structure (see Results). Recordings of poor quality (e.g. too low light intensity or no fish in picture) were discarded from further analysis. An average of the observed water current velocities between surface and 12 m of depth was used in the analysis. Inherently, this type of time series data produces temporal pseudo replication. The relationships between current velocity and observed swimming structure were therefore investigated using mixed effects models to resolve the non-indecencies in our data [21], with swimming structure as fixed effect and time as continuous random effect (function lme, the R software system Version 2.15.0, The R Foundation for Statistical Computing, Vienna, Austria). Model checking plots were used to check that the residuals were well behaved (function plot,∼fitted(.)) and to check the normality assumption (function qqnorm).

## Results and Discussion

**Figure 2:**
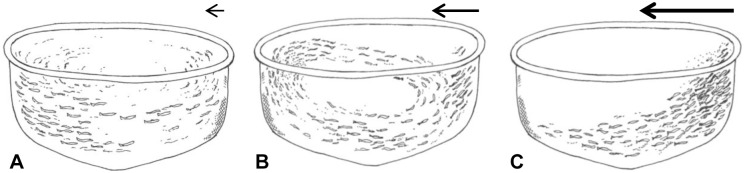
The three observed swimming structures Circle (A, circular movement), Mixed (B, Circle and On Current) or On Current (C, standing on current). The arrows indicate strength and direction of the water current during the different group structures. Drawings by Stein Mortensen, Institute of Marine Research.

A first screening of the videos revealed that the swimming structure could be divided into three main categories: Circle  =  polarized swimming in a circular movement, On Current  =  swimming towards the current with no forward movement and Mixed  =  both Circle and On Current structures present at the same time (Figure 2). Based on data from the more centralised camera (n = 155), the mixed effect model associated the Circle swimming structure with low current velocities (intercept = 22.4 cm s^−1^, SE = 3.1, p<0.001), the Mixed structure with increased current velocities (+13.7 cm s^−1^, SE = 2.2, p<0.001), and the On Current structure with an even higher current velocity (+24.3 cm s^−1^, SE = 1.7, p<0.001). Similarly, for the camera close to the net (n = 347), the Circle structure was associated with low current velocities (intercept = 20.1 cm s^−1^, SE = 2.6, p<0.001) and the Mixed and On Current swimming structures with increasing current velocities (+13.2 cm s^−1^, SE = 2.3, p<0.001 and +26.5 cm s^−1^, SE = 1.4, p<0.001, respectively).

Hence, at low current velocities (≈20 cm s^−1^) the fish swam in circles (Figure 2A, Table 1) and occupied most of the cage volume. With increasing current velocities (≈35 cm s^−1^), a shift occurred with some fish seeking a new position facing the net towards the current while other fish continued to swim in elliptic-shaped circles behind the stationary fish at the net (Figure 2B, Table 1). When the circling fish came to a position where they were exposed to current on their sides they turned inwards toward the centre of the cage and drifted with the current to the leeward side of the cage. Following this, they turned towards the current and continued to swim the remainder of the circle’s distance. With further increase of current velocity, a larger proportion of the group switched from schooling to swimming towards the current next to the net wall, until all fish stood in a dense group along the side of the cage with no circling fish left (≈47 cm s^−1^, Figure 2C, Table 1). With sudden changes of current velocities, there was a period of chaos before the fish established a stable structure again.

**Table 1:** Modeled water current speed in cm**^−^** ^1^ at the reference point for the observed swimming categories Circle (circular movement), Mixed (Circle and On Current) or On Current (standing on current).

Camera pos.	Swimming Structure		
	Circle	Mixed	On Current
Centralized	22.4 (≈0.45)	36.1 (≈0.72)	46.7 (≈0.93)
Side	20.1 (≈0.40)	33.4 (≈0.67)	46.6 (≈0.93)

Water current velocity is given as BL s^−1^ in brackets.

It is thus clear that the fish experience new challenges when exposed to strong water currents. We have for the first time observed shifts in swimming structure of salmon in sea cages connected to changes in current velocities. The shift from the traditional circular schooling to stationary swimming against the current in a group could reflect energetic optimization as a response to the increased current velocities. Fish swimming behind others have been reported to save energy [22]. However, since previously reported U_crit_ [13], [14] is higher than all the observed current velocities this is probably not the only underlying mechanism. The driving force could instead be a combination of U_crit_ and the large variation in current velocities within the cage, thereby restricting the traditional structure when swimming down- compared to upstream.

Theoretically, if a salmon cage is exposed to an increasing current speed, the typical torus shape of a salmon school within the cage will force the upstream fish to double their swimming speed in order to maintain the group structure. If this pattern is broken up by fish changing to stand on the current, the group structure is probable to collapse and move towards a new stable structure with all fish to hold a constant position against the current.

The On Current structure was observed at water current velocities of approximately 47 cm s^−1^ (Table 1). At such velocities fish in a Circle structure swimming against the current would have to swim at least 94 cm s^−1^ to maintain the group structure, which is close to the U_crit_, for Atlantic salmon [13], [14]. However, the dampening effect of the net [20] suggests that a lower current velocity triggers the shift in group structure. Logically, the swimming speed observed in normal schooling structures, during low current velocity, can be identified as the fishes’ preferred speed. This can be termed as such, since fish are able to choose their speed without influence from water current conditions. This chosen speed is assumedly a manifestation of their optimal cruising speed for minimal energy expenditure, as in migrating salmon [10]. Current velocities for Mixed structures were about 35 cm s^−1^ outside the cage; the fish swam in both Circle and On Current structures at this velocity, and this level could represent the approximate breakpoint when the swimming speed started to exceed the preferred swimming speed for some individuals. This current velocity equates to a swimming speed of 2 * 0.7 BL s^−1^ = 1.4 BL s^−1^ (when fish are swimming towards the current in a circular structure), which is higher than previously reported swimming speeds of 0.3–0.9 BL s^−1^ at more sheltered sites [8], [9], [10]. Taking into account the observed dampening effect, the reduced current speed could result in swimming speeds similar to the previously reported preferred swimming speeds.

From a welfare perspective it could be argued that sites with current velocities that do not exceed the school’s preferred swimming speed should provide good welfare since the animal are free to express behaviours within its natural range (item 2 of the Five Freedoms), [23]. Yet, the salmon showed a high degree of plasticity in their behaviour and adapted to the frequent challenges forced upon them by the intermittent and strong water currents. This documented adaptive capacity indicates that conclusions only based on studies performed in laboratories and at unexposed localities could be of limited value due to the different behavioural response to the variable environment. Understanding the effect of water currents on individual fish of different size, as well as on the group as whole, is therefore of utmost importance for the progress of fish farming. High-resolution studies of behaviour in relation to the environment at such sites are needed to ensure environmental conditions acceptable for animal welfare and good production performance.

## References

[1] Holmer M (2010) Environmental issues of fish farming in offshore waters: perspectives, concerns and research needs, Aquac Environ Interact. 1: 57–70.

[2] Perez OM, Telfer TC, Ross LG (2003) On the calculation of wave climate for offshore cage culture site selection: a case study in Tenerife (Canary Islands). Aquac Eng 29: 1–21.

[3] Benetti DD, Benetti GI, Rivera JA, Sardenberg B, O’Hanlon B (2010) Site Selection Criteria for Open Ocean Aquaculture. Mar Technol Soc J 44: 22–35.

[4] Fredheim A, Langan R (2009) Advances in technology for off-shore and open ocean finfish aquaculture. New technologies in aquaculture: improving production efficiency, quality and environmental management. G. Burnell, Allan, G., Woodhead Publishing in Food Science, Technology and Nutrition. 914–944.

[5] Loverich GF, Gace L (1997) The effects of currents and waves on several classes of offshore sea cages. In: Helsley CE, Open Ocean Aquaculture: Charting the Future of Ocean Farming. University of Hawaii, Maui, Hawaii, USA 131–144.

[6] Johansson D, Juell J-E, Oppedal F, Stiansen J-E, Ruohonen K (2007) The influence of the pycnocline and cage resistance on current flow, oxygen flux and swimming behaviour of Atlantic salmon (Salmo salar L.) in production cages. Aquaculture 265: 271–287.

[7] Føre M, Dempster T, Alfredsen JA, Johansen V, Johansson D (2009) Modelling of Atlantic salmon (*Salmo salar* L.) behaviour in sea-cages: A Lagrangian approach. Aquaculture 288: 196–204.

[8] Dempster T, Korsøen O, Folkedal O, Juell JE, Oppedal F (2009) Submergence of Atlantic salmon (*Salmo salar* L.) in commercial scale sea-cages: A potential short-term solution to poor surface conditions. Aquaculture 288: 254–263.

[9] Juell JE (1995) The behaviour of Atlantic salmon in relation to efficient cage rearing. Rev Fish Biol Fish 5: 320–335.

[10] Sutterlin AM, Jokola KJ, Holte B (1979) Swimming behaviour of salmonid fish in ocean pens. J Fish Res Board Can. 36: 948–954.

[11] Brett JR (1964) The respiratory metabolism and swimming performance of young Sockeye salmon. J Fish Res Board Can 21: 1183–1226.

[12] Drenner SM, Clark TD, Whitney CK, Martins EG, Cooke SJ, et al (2012) A synthesis of tagging studies examining the behaviour and survival of anadromous salmonids in marine environments. PloS One 7: 1–13. 10.1371/journal.pone.0031311 PMC3303779 22431962

[13] Deitch EJ, Fletcher GL, Petersen LH, Costa I, Shears MA, et al (2006) Cardiorespiratory modifications, and limitations, in post-smolt growth hormone transgenic Atlantic salmon *Salmo salar* . J Exp Biol 209: 1310–1325. 16547302 10.1242/jeb.02105

[14] McKenzie DJ, Higgs DA, Dosanjh BS, Deacon G, Randall DJ (1998) Dietary fatty acid composition influences swimming performance in Atlantic salmon (*Salmo salar*) in seawater. Fish Physiol Biochem 19: 111–122.

[15] Lijalad M, Powell MD (2009) Effects of lower jaw deformity on swimming performance and recovery from exhaustive exercise in triploid and diploid Atlantic salmon *Salmo salar* L. Aquaculture. 290: 145–154. 10.3354/dao02056 19593934

[16] Stien LH, Bracke MBM, Folkedal O, Nilsson J, Oppedal F, et al.. (2013) Salmon Welfare Index Model (SWIM 1.0): a semantic model for overall welfare assessment of caged Atlantic salmon: review of the selected welfare indicators and model presentation. Rev. Aquaculture: 33–57.

[17] Oppedal F, Dempster T, Stien L (2011) Environmental drivers of Atlantic salmon behaviour in sea-cages: a review. Aquaculture 311: 1–18.

[18] Remen M, Oppedal F, Torgersen T, Imsland AK, Olsen RE (2012) Effects of cyclic environmental hypoxia on physiology and feed intake of post-smolt Atlantic salmon: Initial responses and acclimation. Aquaculture 326–329: 148–155.

[19] Gansel LC, McClimans TA, Myrhaug D (2012) Flow around the free bottom of fish cages in a uniform flow with and without fouling. J Offshore Mech Arct Eng 134, DOI: 10.1115/1.4003695.

[20] Klebert P, Gansel L, Lader P, Oppedal F (2013) Flow hydrodynamics through nets and floating cages: a review. Ocean Eng 58: 260–274.

[21] Crawley MJ (2007) Mixed-effects models, in: The R Book. West Sussex, Wiley 627–660.

[22] Herskin J, Steffensen JF (1998) Energy savings in sea bass swimming in a school: measurements of tail beat frequency and oxygen consumption at different swimming speeds. J Fish Biol 53: 366–376.

[23] Electronic reference: UK Farm Animal Welfare Council (2005) The Five Freedoms. Available: http://www.fawc.org.uk/freedoms.htm. Accessed 2013 Apr 11.

